# 

**Published:** 1899-02-18

**Authors:** 


					CADBURY'S O CADBURY'S
COCOA
NO DRUGS OR ALKALIES USED.
^647. Vol, XXV. Satttbday,
Feb. 18th, 1899.
5 tliMtUL&AUJaWii men HOSBWAL
["Subscription Terms,-!
L see p. 812,
PRICE TWOPENCE.

				

